# Molecular characterization of extended-spectrum β-lactamase-producing *Escherichia coli* isolated from postpartum uterine infection in dairy cattle in India

**DOI:** 10.14202/vetworld.2021.200-209

**Published:** 2021-01-23

**Authors:** Samiksha Agrawal, Ajay Pratap Singh, Rashmi Singh, Raktim Saikia, Soumen Choudhury, Amit Shukla, Shyama N. Prabhu, Jitendra Agrawal

**Affiliations:** 1 College of Biotechnology, Uttar Pradesh Pandit Deen Dayal Upadhyaya Pashu Chikitsa Vigyan Vishwavidhyalaya Ewam Gau Anusandhan Sansthan Mathura, Uttar Pradesh, India; 2 Department of Veterinary Microbiology, Uttar Pradesh Pandit Deen Dayal Upadhyaya Pashu Chikitsa Vigyan Vishwavidhyalaya Ewam Gau Anusandhan Sansthan Mathura, Uttar Pradesh, India; 3 Department of Veterinary Pharmacology, Uttar Pradesh Pandit Deen Dayal Upadhyaya Pashu Chikitsa Vigyan Vishwavidhyalaya Ewam Gau Anusandhan Sansthan Mathura, Uttar Pradesh, India; 4 Department of Veterinary Pathology, Uttar Pradesh Pandit Deen Dayal Upadhyaya Pashu Chikitsa Vigyan Vishwavidhyalaya Ewam Gau Anusandhan Sansthan Mathura, Uttar Pradesh, India; 5 Department of Animal Reproduction and Gynecology, College of Veterinary Science and Animal Husbandry, Uttar Pradesh Pandit Deen Dayal Upadhyaya Pashu Chikitsa Vigyan Vishwavidhyalaya Ewam Gau Anusandhan Sansthan Mathura, Uttar Pradesh, India

**Keywords:** antibiotic resistance, cattle, endometritis, extended-spectrum β-lactamases, microbiology

## Abstract

**Background and Aim:**

Selection and dissemination of plasmid-encoded extended-spectrum β-lactamase (ESBL) among *Enterobacteriaceae* confers resistance to beta-lactam antibiotics. The purpose of this study was to determine the prevalence and molecular characteristics of ESBL-producing organisms isolated from dairy cattle with a uterine infection.

**Materials and Methods:**

Bacterial isolates (n=62) were characterized by biochemical test for genus and species determination. Antimicrobial susceptibility tests were performed by Kirby–Bauer disk diffusion method using panel of antibiotics for initial screening of ESBL organism. Phenotypic confirmation of ESBL-suspected strains was done by combination disk method and double-disk method. Multiplex polymerase chain reaction (PCR) was carried out for phylogrouping of *Escherichia coli* isolates as well as for genotyping ESBL genes. Enterobacterial repetitive intergenic consensus-PCR method was used for genotypic characterization of isolates.

**Results:**

Antibiotic susceptibility profile of *E. coli* (n=40) isolates showed high rates of resistance for ampicillin (95.0%), cefpodoxime (97.5%), cefotaxime (87.5%), and ceftriaxone (70%). However, low rates of resistance were observed for cefoxitin (25%), amoxicillin/clavulanic acid (20%), ceftazidime (17.5%), gentamicin (10%), and ertapenem (7.5%). A total of 39/40 *E. coli* isolates were confirmed as ESBL with Epsilometer test as well as the genotypic method and 28 (70%) of them were multidrug-resistant. Genotype *bla_CTX-M_* was observed as a predominant beta-lactamase type with the preponderance of CTX-M Group 1. The following combinations were observed: *bla_TEM_*+ *bla_CTX-M_* in 15 (36.2%) isolates, *bla_TEM_*/*bla_SHV_* in 8 (5.2%) isolates, and *bla_CTX-M_*/*bla_SHV_* in 6 (5.2%) isolates. The phylogenetic grouping of *E. coli* strains revealed the highest prevalence for B1 (22.0%) followed by A (20%).

**Conclusion:**

This report shows a high frequency of ESBL *E. coli* from cattle with postpartum uterine infections. These isolates showed reduced susceptibility to common antibiotics used for the treatment of uterine infections greater affecting the therapeutic outcome.

## Introduction

India is home to the world’s largest bovine population which contributes to ~19% of the global milk production in the world [1]. Postpartum uterine infections are one of the most important health-related problems in bovine causing significant economic losses to the dairy farmers due to the culling of the infertile animal, lower milk yield, and the rising cost of treatment [2]. Several different species of pathogens are known to be associated with clinical endometritis and infertility; however, the role of *Escherichia coli* has been stressed by several studies [3-7]. The traditional method for the treatment of bovine endometritis mainly involves the therapeutic use of antibiotics [8]. However, in recent years, the antimicrobial therapy of endometritis has resulted in higher proportions of treatment failures with inconsistent recovery rate. The attenuated effect of the therapeutic efficacy of antibiotics is largely contributed by the emergence of antimicrobial resistance [9].

Production of extended-spectrum β-lactamases (ESBLs) is the most frequent cause of resistance to b-lactam antibiotics in Gram-negative bacteria. ESBL genes generally carried on mobile genetic elements that facilitate its spread at fast rates among commensal and pathogenic bacteria in the herd and the environment. ESBL enzymes hydrolyze penicillins, cephalosporins, and the monobactam aztreonam rendering them ineffective. ESBL production is mainly mediated by *bla_TEM_*, *bla_SHV_*, and *bla_CTX-M_* genes [10,11]. The CTX-M β-lactamases can be divided into five sublineages or groups: The CTX-M Group 1; CTX-M Group 2, CTX-M Group 8, CTX-M Group 9, and CTX-M Group 25 [12]. Recently, CTX-M has emerged as the most dominant genotype in Asia with the highest relative abundance of *bla_CTX-M_* Group 1 gene [13]. The ESBL-producing *E. coli* is an important constituent of the fecal flora of livestock, which may serve as a reservoir and source of transmission to humans by various direct and indirect means [14,15]. Recently, many studies have reported ESBL-producing *E. coli* isolated from cattle [16], poultry [17], and pigs [18]. Recent surveys suggest widespread colonization of ESBL *Enterobacteriaceae* in food-producing animals [19]. In India, ESBL-producing *E. coli* are mostly reported in food-producing animals and their environment through milk, meat, fecal samples, and bovine mastitis [17,20-25]. Metritis and endometritis are the major postpartum clinical conditions in bovine, characterized by inflammation of the uterus. Endometritis is an important contributor to bovine infertility extending the calving to conception interval, increasing the number of services per conception and the proportion of culls for failure to conceive.

Although there is growing a sense of urgency in reporting the prevalence of ESBLs from livestock in India, most of the studies are incoherent. Moreover, the epidemiological studies regarding the prevalence of ESBL-producing bacteria in bovine uterine infections are largely ignored. In this study, we report the prevalence of ESBL-producing *E. coli* in the uteri of cows with endometritis and further characterized them based on phenotypic and genotypic tests.

## Materials and Methods

### Ethical approval

This study was approved by the Institutional Animal Ethics committee of College of Veterinary Sciences, Uttar Pradesh Pandit Deen Dayal Upadhyaya Pashu Chikitsa Vigyan Vishwavidyalaya Evam Go Anusandhan Sansthan (DUVASU), Mathura, India with ref No. IAEC/18/23.

### Study location, period and sample collection

Samples were collected from cases presented to the Veterinary Clinical Complex, College of Veterinary Sciences, DUVASU, Mathura, from May 2018 to September 2018. Puerperal metritis was diagnosed based on clinical observation such as an abnormally enlarged uterus and a fetid, watery, red-brown uterine discharge. The uterine discharge was obtained from each animal using a sterile swab by the double guarded method to prevent vaginal contamination. Each swab was transferred to a 20 mL collection vial containing 10 mL buffered peptone water, immediately placed on ice, and transported to the laboratory. Samples were incubated overnight at 37°C before inoculation.

### Isolation and identification of bacterial strains

The pre-enriched samples were streaked onto MacConkey agar (Sigma-Aldrich) supplemented with cefotaxime and ceftazidime (HiMedia, India) at the concentration of 2 μg/mL each and incubated at 37°C for 18-24 h. One colony from each type was picked from primary growth and subcultured on the MacConkey agar plate supplemented with both cefotaxime and ceftazidime at the final concentration of 1 μg/mL. Purified isolates were identified using the procedures described by Cowan and Steel [26]. Biochemically confirmed isolates were stored in trypticase soy broth (TSB; Sigma-Aldrich) containing 30% glycerol at −80°C.

### Antibiotic susceptibility test

Antimicrobial susceptibility test was performed for all *E. coli* isolates by standard Kirby–Bauer disk diffusion method using Mueller-Hinton agar (Sigma-Aldrich, USA) as per Clinical and Laboratory Standards Institute guidelines [27]. Briefly, three to four well-isolated colonies from overnight grown culture plates were transferred to sterile saline with a loop. The bacterial suspension was adjusted to give turbidity equivalent to 0.5 McFarland standards corresponding to 1×10^8^ CFU/mL. A total volume of 1 mL of standardized inoculums were spread onto Mueller-Hinton agar plate and antimicrobial-impregnated disks (Becton Dickinson, Sparks, MD, USA) were placed. The used antibiotics were ertapenem (10 μg), cefotaxime (30 μg), ceftazidime (30 μg), gentamicin (10 μg), ampicillin (10 μg), amoxicillin-clavulanate (10 μg), ciprofloxacin (5 μg), cefoxitin (30 μg), ceftriaxone (30 μg), and cefpodoxime (10 μg). Zone of inhibition was measured in mm and interpreted as sensitive, intermediate, or resistant. *E. coli* ATCC 25922 (ESBL-negative strain) and *Klebsiella pneumoniae* ATCC 700603 (ESBL-positive strain) were used as control strains (Table-1). Multiple antibiotic resistance (MAR) index was determined for each isolate using the formula MAR=a/b, where a represents the number of antibiotics to which the test isolate depicted resistance and b represents the total number of antibiotics to which the test isolate has been evaluated for susceptibility.

**Table-1:** AST zone diameters for control strain *E. coli* (ATCC^®^25922™) and the test *E. coli* isolates* used in this assay.

Antibiotic	Disk code	Antibiotic concentration (µg)	Control strain zone diameter range (mm)	Control strain diameter observed (mm)	Test zone diameters (mm)		

					Resistant	Intermediate	Susceptible
Amoxicillin/clavulanic acid	AmC-30	20/10	18-24	22	≤13	14-17	≥18
Ampicillin	AM-10	10	16-22	20	≤13	14-16	≥17
Cefotaxime	CTX-30	30	29-35	34	≤14	15-22	≥23
Ceftazidime	CAZ-30	30	25-32	29	≤14	15-17	≥18
Cefpodoxime	CPD-10	10	23-28	25	≤17	18-20	≥17
Ceftriaxone	CRO-30	30	29-35	29	≤13	14-20	≥21
Cefoxitin	FOX-30	30	23-29	24	≤14	15-17	≥18
Ciprofloxacin	CIP-5	5	30-40	30	≤15	16-20	≥21
Gentamicin	GM-10	10	19-26	17	≤12	13-14	≥15
Ertapenem	ETP-10	10	29-36	32	≤15	16-18	≥19

* Adapted from M100, Performance Standards for AST; 27^th^ edition, (Published by Clinical and Laboratory Standards Institute, CLSI 2017, Pennsylvania, USA. AST=Antimicrobial susceptibility testing, *E. coli=Escherichia coli*

### Phenotypic detection of ESBL production

All *E. coli* isolates suspected of producing ESBL based on zone diameter breakpoints corresponding to resistant phenotype were included in the study. All the ESBL-suspected isolates were grown on trypticase soy agar (TSA, Sigma-Aldrich) for an overnight incubation, before phenotypic testing by combination disk method, double-disk diffusion method, and ESBL MIC reduction E-test as previously described or with some modifications. *E. coli* ATCC 25922 (ESBL-negative strain) and *K. pneumoniae* ATCC 700603 (ESBL-positive strain) were used as control strains.

### Analysis of antimicrobial resistance genes

The DNA extraction was performed using heat lysis (Snap-chill method) method. Briefly, loopful of bacterial culture was suspended with 200 μL nuclease-free water thoroughly in microcentrifuge tube followed by heating in a boiling water bath for 10 min. Boiled cell lysate was centrifuged at 12,000 rpm for 2 min. Three microliters of supernatant were used as a template for polymerase chain reaction (PCR) reaction. DNA isolated was subjected to a target amplification of ESBL-associated genes encoding CTX-M, TEM, and SHV using previously described oligonucleotide primers as described in Table-2. Multiplex PCR was also performed for the identification of CTX-M genes using specific primers designed for identifying CTX-M groups (individually, CTX-M Groups 1, 2, and 9, and, together, Groups 8 and 25). A 25 μL reaction mixture containing 12.5 μL DreamTaq Master Mix, a variable concentration of specific group primers (Table-2) and 2 μL of isolated DNA template were used. Amplification was carried involving initial denaturation at 94°C for 10 min and 30 cycles of denaturation at 94°C for 40 s, at variable annealing temperature for each primer (Table-2) for 40 s, and extension at 72°C for 1 min followed by final elongation step at 72°C for 7 min. Amplicons were visualized after running at 80 V for 2 h on 2% agarose gel containing ethidium bromide (0.5 μg/mL) with a 100 bp DNA ladder as a size marker. *K. pneumonia* ATCC 700603 (ESBLs positive strain) was used as a positive control.

**Table-2:** Details of primers used in the study.

PCR name	Primer name	Sequence (5’-3’)	Primer concentration (pmol)	Tm for each PCR reaction	Amplicon size	References
CTX-M universal primer	CTX-M universal_F	CGA TGT GCA GTA CCA GTA A	15	57°C	580	[29]
	CTX-M universal_R	TTA GTG ACC AGA ATC AGC GG	15	57°C		
Multiplex I TEM and SHV	MultiTSO-T_for	CATTTCCGTGTCGCCCTTATTC	20	60°C	800	[30]
	MultiTSO-T_rev	CGTTCATCCATAGTTGCCTGAC	20	60°C		
	MultiTSO-S_for	AGCCGCTTGAGCAAATTAAAC	20	60°C	713	
	MultiTSO-S_rev	ATCCCGCAGATAAATCACCAC	20	60°C		
Multiplex II CTX-M Group 1, Group 2, and Group 9	MultiCTXMGp1_for	TTAGGAARTGTGCCGCTGYAb	20	60°C	688	
	MultiCTXMGp1-2_rev	CGATATCGTTGGTGGTRCCATb	10	60°C		
	MultiCTXMGp2_for	CGTTAACGGCACGATGA	10	60°C	404	
	MultiCTXMGp2_rev	CGATATCGTTGGTGGTRCCATb	10	60°C		
	MultiCTXMGp9_for	TCAAGCCTGCCGATCTGGT	20	60°C	561	
	MultiCTXMGp9_rev	TGATTCTCGCCGCTGAAG	20	60°C		
CTX-M Group 8/25	CTX-Mg8/25_for	AACRCRCAGACGCTCTACb	10	60°C	326	
	CTX-Mg8/25_rev	TCGAGCCGGAASGTGTYATb	10	60°C		
Multiplex phylogrouping assay	chuA.1b	ATGGTACCGGACGAACCAAC	20	59°C	288	[28]
	chuA.2	TGCCGCCAGTACCAAAGACA	20	59°C		
	yjaA.1b	CAAACGTGAAGTGTCAGGAG	20	59°C	211	
	yjaA.2b	AATGCGTTCCTCAACCTGTG	20	59°C		
	TspE4C2.1b	CACTATTCGTAAGGTCATCC	20	59°C	152	
	TspE4C2.2b	AGTTTATCGCTGCGGGTCGC	20	59°C		
	AceK.f	AACGCTATTCGCCAGCTTGC	40	59°C	400	
	ArpA1.r	TCTCCCCATACCGTACGCTA	40	59°C		
	ArpAgpE.f	GATTCCATCTTGTCAAAATATGCC-	20	57°C	301	
	ArpAgpE.r	GAAAAGAAAAAGAATTCCCAAGAG-	20	57°C		
	trpAgpC.1	AGTTTTATGCCCAGTGCGAG	20	59°C	219	
	trpAgpC.2	TCTGCGCCGGTCACGCCC	20	59°C		
	trpBA.f	CGGCGATAAAGACATCTTCAC	40	59°C	489	
	trpBA.r	GCAACGCGGCCTGGCGGAAG	40	59°C		
ERIC-PCR	ERIC-1R	AAG CTC CTG GGG ATT CA	50	50°C		[31]
	ERIC-2	AAGTAAGTGACTGGG GTGAGCG	50	50°C		

ERIC-PCR=Enterobacterial repetitive intergenic consensus-polymerase chain reaction

### Phylogenetic determination of β-lactamase-producing E. coli isolates

A quadruplex PCR assay targeting the *chuA* and *yjaA* genes, *TspE4* and *arpA*, and a simplex PCR using Group E and Group C specific primers using trpA as an internal control was carried out as proposed by Clermont *et al*. [28]. The primers sequences and products size are listed in Table-2 [28-31]. PCR reactions were performed under the following condition: Denaturation at 94°C for 4 min, 30 cycles of 5 s at 94°C and 20 s at 57°C (Group E) or 59°C (quadruplex and Group C), and a final extension at 72°C for 5 s followed by final elongation step at 72°C for 7 min.

### Enterobacterial repetitive intergenic consensus (ERIC)-PCR

ERIC-PCR reactions were performed in 20 μL volumes containing 50 pmol of each primer (final concentration), 10 μL of the master mix, 1.5 μL of MgCl_2_, 2 μL of template DNA, and 3.5 μL of deionized water. PCR amplification was achieved by initial denaturation at 94°C for 30 s, annealing at 50°C for 45 s, and extension at 72°C for 3 min followed by final elongation step at 72°C for 7 min. PCR products were electrophoresed using 1.5% agarose (Puregene) gel electrophoresis at 80 V for 180 min then visualized under UV in a gel documentation system. The image was analyzed by GelJ software (https://sourceforge.net/projects/gelj/).

## Results

### Endometritis-associated uterine Enterobacteriaceae

Uterine sampling of cows (n=34) and buffaloes (n=36) with clinical endometritis identified a total of 62 bacterial isolates. The isolates are distributed as follows: *E. coli* was found to be the most common identified *Enterobacteriaceae*, with occurrence of 64.5% (n=40), *Klebsiella* spp. 11.2% (n=7), *Citrobacter* spp. 8.06% (n=5), *Serratia* spp. 6.45% (n=4), and *Enterobacter* spp. 3.22% (*n*=2). Among non-fermentative Gram-negative bacilli, *Pseudomonas* spp. 3.22% (*n*=2) was recovered. Two isolates could not be identified by the biochemical tests.

### Antibiotic susceptibility

**Figure-1:**
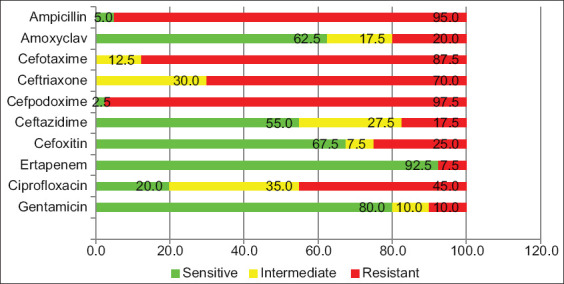
Percentage of uterine isolates indicating resistance, intermediate resistance, and sensitive pattern for 10 different antibiotics.

**Figure-2:**
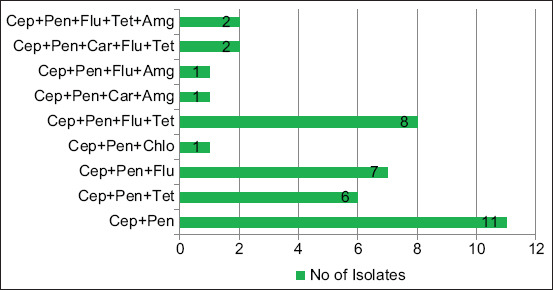
Antimicrobial resistance profile of Escherichia coli isolates obtained from infected bovine uterus Amg= Aminoglycoside, Car=Carbapenam, Cep= Cephalosporin, Chlo=Chloramphenicol, Flu= Fluoroquinolones, Pen= Penicillin, Tet= Tetracycline.

The results of antibiotic susceptibility of the 40 *E. coli* isolates are presented in Figure-1. The majority of the isolates were resistant to several beta-lactam antimicrobials including ampicillin (95.0%), cefpodoxime (97.5%), cefotaxime (87.5%), and ceftriaxone (70%). All the isolates (100%) were found resistant to at least one of the three cephalosporin antibiotics (cefotaxime, ceftriaxone, and cefpodoxime), while 70% (n=28) were resistant to the entire three cephalosporin antibiotics. However, a high rate of susceptibility was observed toward ceftazidime (82.5%), amoxiclav (80%), and cefoxitin (75%). The isolates showed medium to a low level of resistance to non-b-lactam antibiotics including ciprofloxacin (45%) and gentamicin (10%). Resistance to ertapenem was observed in 7.5% of the isolates. Of the 40 *E. coli* isolates, 70% were multidrug-resistant (MDR) and showed resistance to three or more different antibiotic groups (Figure-2). The mean MAR index for *E. coli* isolates was 0.47 (range 0.3-0.86).

### Phenotypic and genotypic characterization ESBL E. coli

**Figure-3:**
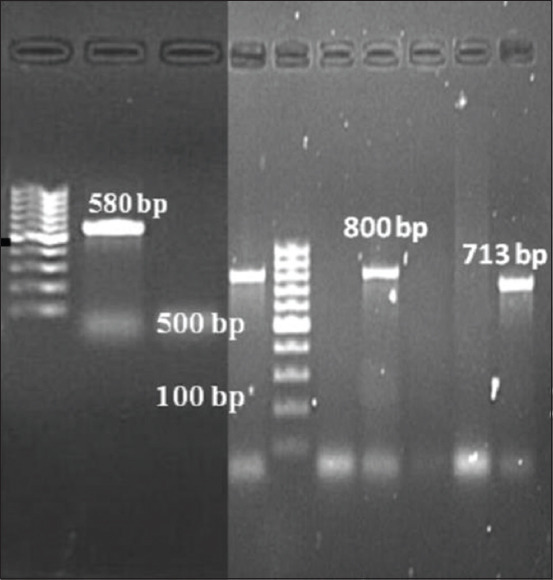
Polymerase chain reaction amplification of *bla_CTX-M_* gene (580 bp), *bla_TEM_* (800 bp), and *bla_SHV_* (713 bp) gene, lane M: 100 bp DNA ladder, lane 1: *bla_CTX-M_*, lane 3,6: *bla_TEM_* isolate, lane 9 *bla_SHV_*, and lane 2 and 8 : *Escherichia coli* ATCC 25922.

**Figure-4:**
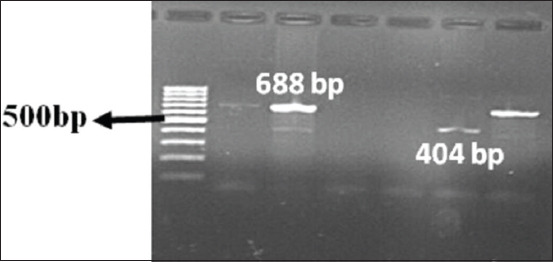
Polymerase chain reaction amplification of *bla_CTX-M_* Group 1 and Group 2 gene, lane M: 100 bp DNA ladder, lane 1-6: *bla_CTX-M_* Group 1, lane 5 bottom row: *bla_Ctx-M_* Group 2.

Out of 40 *E. coli* isolates, 31 (77.5%) were phenotypically identified as presumptive ESBL producers. The ESBL E-test could confirm all the 40 isolates (100%) as ESBL producers. Therefore, all *E. coli* isolates were genotyped using multiplex PCR (Figures-3 and 4). ESBL genotype-specific PCR assay could detect 39 (97.5%) as ESBL producers. The overall prevalence of *bla_CTX-M_*, *bla_TEM_*, and *bla_SHV_* genes among ESBL *E. coli* isolates recorded were 84.5%, 37.9%, and 5.2%, respectively. The coexistence of two of the genes was observed at the highest range with *bla_TEM_* and *bla_CTX-M_* in 15 (36.2%) isolates followed by *bla_TEM_* and *bla_SHV_* in 8 (5.2%) isolates and *bla_CTX-M_* and *bla_SHV_* in 6 (5.2%) isolates. Notably, one isolate carried the three β-lactamase genes. Multiplex PCR for subgrouping of *bla_CTX-M_* revealed 77.5% (n=31) isolates was positive for *bla_CTX-M_* Group 1 while 1 (2.5%) was having amplicon specific for *bla_CTX-M_* Group 2. For eight isolates, multiplex PCR for CTX-M grouping fails to give any result.

### Phylogrouping of E. coli isolates

All *E. coli* isolates were allocated to one of the eight phylogenetic groups (A, B1, B2, D, F, E, C, and Clad 1) according to Clermont*et al*. [28]. The results showed that 22.5% (n=9) of these isolates belonged to phylogroup B1, 20% (n=8) Group A, 12.5% (n=5) each for Groups C and D, 7.5% (n=5) Group F, and 2.5% (n=1) each for Group E and Clad I.

### ERIC-PCR

**Figure-5:**
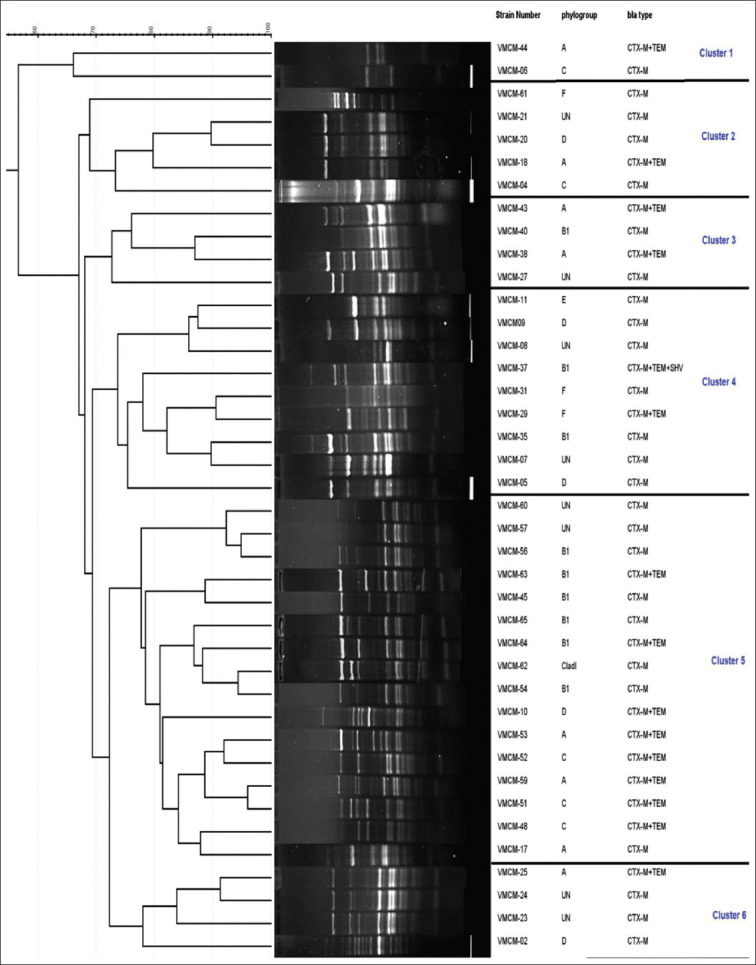
Dendrogram of enterobacterial repetitive intergenic consensus-polymerase chain reaction pattern of extended-spectrum β-lactamase *Escherichia coli* strains. The analysis of similarity of the normalized images was accomplished using UPGMA method with arithmetic mean with Dice optimized coefficient.

The clonal relatedness analysis of the 40 *E. coli* isolates was carried out by the ERIC-PCR fingerprint method. Eight (20%) isolates were considered non-typeable by ERIC-PCR. ERIC-PCR generated several amplified products ranging from ~150 bp to >1000 bp. The isolates produced different DNA profiles by ERIC-PCR ranging from 1 to 15 bands. Several bands were consistently present in all isolates showing clonal relatedness. The dendrogram was generated after analysis with GelJ software using UPGMA from the ERIC-PCR (Figure-5). Based on clustering, isolates could be grouped into six mini-clusters. Detailed analysis of bacterial strains from different clusters revealed that strains distributed across the cluster were genetically heterogeneous. No direct correlation whatsoever could be established in the strains clustered together regarding species (cow or buffalo), age, parity of animals, and beta-lactamase gene type. However, it was noticed that most of the isolates with resistant profile Cep^+^Pen^+^Tet^+^ variants were clustered in clusters 2 and 3, while clusters 4, 5, and 6 predominately have Cep^+^Pen^+^Tet^+^ Flu^+^ resistance profile.

## Discussion

Even though *E. coli* has been described as a normal inhabitant of the bovine reproductive tract; their role as a major pathogen in clinical endometritis is unchallenged. Beta-lactam antibiotics, especially ceftiofur (third-generation cephalosporin), are currently the treatments of choice for uterine infections in cattle. Extensive use of beta-lactam antibiotics is one of the major attributes for the emergence and spread of ESBL-producing bacterial strains in the recent past. ESBL enzymes confer resistance to extended-spectrum cephalosporins and have an attenuated effect of antibiotic treatment and a high rate of treatment failure. There is a complete lack of scientific information regarding the reports and data reflecting the prevalence of ESBL organisms from bovine uterine infection in India. This study provides information regarding the prevalence of ESBL-producing organisms in the infected uterus of bovines with clinical endometritis and highlights the role of ESBL-producing *E. coli* in the pathogenesis of endometritis.

In this study, bacteria isolated from the uterus of infected postpartum cows and buffaloes demonstrated the dominance of *E. coli*. The present finding extends support to the previous observation of *E. coli* as being the most prevalent organism in bovine, suffering from post-parturient reproductive disorders [32-35]. *E. coli* commonly found in the gut and integument of animals and the environment. At parturition, massive contamination with these opportunistic organisms in the genital tract leads to endometritis and infertility.

High frequency of resistance against penicillin and cephalosporin class among ESBLs in the current study was in accordance with the similar observation made by others [13,36]. Of the 40 isolates, close to 98% isolates were found to be resistant to at least one of the four cephalosporin antibiotics tested (cefotaxime, ceftriaxone, and cefpodoxime), while 70% were resistant to all three cephalosporin antibiotics. The antibiogram observed in the current study reflects the findings of other researchers; however, unlike other studies, we have observed a high level of sensitivity to amoxicillin-clavulanic acid and ceftazidime [37]. It was indicated that 45% of ESBL-producing isolates were also ciprofloxacin-resistant. Cross-resistance with other groups of antibiotics such as fluoroquinolones has been more commonly observed from ESBL-producing bacteria [38].

A perusal of antibiotic susceptibility test result showed that 70% (n=28) isolates were MDR. This finding correlates with similar results obtained from a study conducted by Sáenz *et al*. [39] that have reported similar antibiotic resistance profile by *E. coli* isolated from cattle. Ibrahim *et al*. [36] showed 92% resistance to at least one antibiotic, 57.9% showed resistance to three or more antibiotics from three different antibiotic groups. Over recent years, several such studies have highlighted the occurrence of MDR ESBL-producing *E. coli* isolated from several livestock sources including pigs, poultry, and cattle [16-18].

The prevalence of ESBL-producing *E. coli* in India has noticeably increased in recent years in animals and associated environment [40]. In our study, the overall percentage of ESBL-producing *E. coli* isolates was 64.5% (according to the number of investigated strains), at rates similar to those previously reported from other livestock sources [20-25,41]. We compared PCR-based ESBL detection test with ESBL E-test and double-disk synergy test, with regard to their accuracy and effectiveness in detecting ESBL production in *E. coli*. While ESBL E-test could able to detect 100% isolates as ESBL, 97.5% isolates were confirmed by the genotypic method as ESBL positive. Positional changes within the beta-lactamase genes are frequently observed to give rise to the variants. These variants are frequently not detected by standard PCR. Double-disk method and combination disk tests are the least accurate as it could able to detect only 77.5% isolates as ESBL in the present study. A high rate of detection of ESBL by the E-test could be due to the inclusion of false-positive results. Färber *et al*. [42] reported 6% false-positive results for ESBL E-test in comparison with ESBL-positive strains confirmed by the genotypic method. Notably, the diagnostic accuracy ESBL E-test is only 94% when compared with that of the molecular identification method of ESBL production [43]. PCR-based identification of beta-lactamase genes is the most reliable method to identify ESBL-producing *Enterobacteriaceae*. However, its amalgamation into the routine diagnostic process remains a challenge due to high cost and labor intensiveness.

The most frequent ESBLs in *Enterobacteriaceae* belong to the TEM, SHV, and CTX-M families. The present study indicates high carriage rates of *bla_CTX-M_* type ESBL (84.5%) among uterine isolates with *bla_CTX-M_* Group 1 being the major (77.5%) allele encoding for ESBLs. The findings of this research concur with previous studies around the world [14,15,17,44]. In South Asia, 85-90% of ESBLs carry *bla_CTX-M_* genotypes [45,46]. Of the *bla_CTX-M_* genes, those belonging to the *bla_CTX-M_* Group 1 are the most widespread genes encoding for ESBLs [13,40].

Most of *E. coli* isolates can be categorized into several phylogenetic groups, namely, A, B1, B2, C, D, E, and F [47]. The previous findings are compatible with the results of our study in which *E. coli* isolates associated with clinical endometritis mainly belonged to phylogroup A, while phylogroup B2 was not detected [48-51]. Phylogenetic groups A and B1 were mostly associated with commensal and diarrheagenic strains, whereas the close association of phylogenetic groups B2 and D was established in extraintestinal infections and invasive strains [52]. Hence, the colonization of phylogenetic groups A and B1 in the uterus can be corroborated with the fact that postpartum fecal contamination of uterus remains the most remarkable cause of clinical endometritis. ERIC-PCR analysis of *E. coli* isolates revealed that, nevertheless, the isolates were from a narrow geographical boundary that the strains were genetically heterogeneous. From this study, we observe that there is no correlation of the clonally related strains with their respective phylogroup, the pattern of the beta-lactamase gene content. Several isolates showed common banding pattern indicating the dissemination of clonally related organisms between the host animals. Antibiotic selection pressure could be the most important attribute for the spread and occurrence of clonally similar groups among these isolates in the absence of correlation with their respective antibiogram or with the type of beta-lactamases gene they harbor. Although ERIC profiling reflects true genetic relatedness of a group of bacterial isolates, its usefulness for molecular epidemiological investigation of antimicrobial-resistant isolates holds limited value. Despite the fact that ERIC-PCR demonstrates reasonable high level of concordance, discrimination, and typeability, pulse-field gel electrophoresis and multilocus sequence typing are better suited for inferring relatedness among isolates.

## Conclusion

The present study highlights a high prevalence of ESBL-producing *E. coli* associated with postpartum uterine infections in bovine. A high level of resistance was observed for the third-generation cephalosporin, which is the treatment of choice for such infection in bovines. The colonization of these bacteria significantly affects the clinical outcome of treatment using cephalosporin antibiotics and warrants an alternate line of treatment.

## Authors’ Contributions

SA, APS, and RS contributed to the study conception. SA, RaS, and JA contributed in sample collection. SC and RS designed and conducted the experiments. APS and SNP analyzed the data. APS drafted the manuscript. AS edited the manuscript. All authors read and approved the final manuscript.
